# HALT (Hernia Active Living Trial): protocol for a feasibility study of a randomised controlled trial of a physical activity intervention to improve quality of life in people with bowel stoma with a bulge/parastomal hernia

**DOI:** 10.1186/s40814-020-00674-2

**Published:** 2020-09-24

**Authors:** Gill Hubbard, Rebecca J. Beeken, Claire Taylor, Raymond Oliphant, Angus J. M. Watson, Julie Munro, Sarah Russell, William Goodman

**Affiliations:** 1 Department of Nursing and Midwifery, University of the Highlands and Islands, Centre for Health Science, Old Perth Road, Inverness, IV2 3JH UK; 2 Leeds Institute of Health Sciences, University of Leeds, Worsley Building, Clarendon Way, Leeds, LS2 9NL UK; 3 St Mark’s Hospital, London North West University Healthcare NHS Trust, Harrow, Middlesex, HA1 3UJ UK; 4 Department of Surgery, Raigmore Hospital, NHS Highland, Old Perth Rd, Inverness, IV2 3UJ UK; 5 Farmside House, Tidebrook, East Sussex, TN5 6PE UK; 6 Research Department of Behavioural Science and Health, University College London, Gower Street, London, WC1E 6BT UK

**Keywords:** Parastomal hernia, Colostomy, Ileostomy, Bowel disease, Feasibility study

## Abstract

**Background:**

Parastomal hernia (PSH) can be repaired surgically, but results to date have been disappointing, with reported recurrence rates of 30 to 76%. Other types of intervention are therefore needed to improve the quality of life of people with PSH. One potential intervention is physical activity. We hypothesise that the intervention will increase core activation and control across the abdominal wall at a site of potential weakness and thus reduce the risk of PSH progression. Increases in physical activity will improve body image and quality of life (QoL).

**Methods:**

Subjects and sample

There were approximately 20 adults with a bowel stoma and PSH. People with previous PSH repair will be excluded as well as people who already do core training.

Study design

This is a feasibility study of a randomised controlled trial with 2 months follow-up, in 2 sites using mixed methods. Stage 1 involves intervention development and in stage 2, intervention and trial parameters will be assessed.

Intervention

A theoretically informed physical activity intervention was done, targeting people with PSH.

Main outcome of feasibility study

The main outcome is the decision by an independent Study Steering Committee whether to proceed to a full randomised controlled trial of the intervention.

Other outcomes

We will evaluate 4 intervention parameters—fidelity, adherence, acceptability and safety and 3 trial parameters (eligible patients’ consent rate, acceptability of study design and data availability rates for following endpoints):

I. Diagnosis and classification of PSH
II. Muscle activation
III. Body composition (BMI, waist circumference)
IV. Patient reported outcomes: QoL, body image and physical functioning
V. Physical activity;
VI. Psychological determinants of physical activity

Other data

Included are other data such as interviews with all participants about the intervention and trial procedures.

Data analysis and statistical power

As this is a feasibility study, the quantitative data will be analysed using descriptive statistics. Audio-recorded qualitative data from interviews will be transcribed verbatim and analysed thematically.

**Discussion:**

The feasibility and acceptability of key intervention and trial parameters will be used to decide whether to proceed to a full trial of the intervention, which aims to improve body image, quality of life and PSH progression.

**Trial registration:**

ISRCTN15207595

## Background

### Parastomal hernia

Parastomal hernia (PSH) is a common problem following stoma formation [1, 2], with prevalence estimates over 30% by 12 months, 40% by 2 years and 50% or higher at longer duration of follow-up [3]. A cross-sectional study of 75 patients with an end colostomy for ≥ 1 year found that 33 (44%) had evidence of a PSH on computerised tomography (CT) [4]. The majority of these patients were symptomatic (27 versus 6), such as pain or difficulties with stoma appliance and able to identify the moment of clinical appearance of the PSH, which occurred within 8 months of surgery [4].

One of the functions of deep abdominal muscles is to provide support to the abdominal region and the spine by forming a muscle band that tightens like a corset [5]. Following abdominal surgery, the physiology of the abdominal wall is altered with damage to nerve supply and atrophy of the midline muscular wall [6]. Surgery for creating a stoma alters the physiology in the same way and creates a further site of weakness by leaving a hole in the abdominal wall. Evidence indicates that there is muscular atrophy directly below the stoma site, resulting in change of forces and pressure on the abdominal wall [7].

There is a paucity of prospective data about the natural history and trajectory of PSH and whether PSH severity progression can be arrested [8, 9]. PSH can be repaired surgically but results to date have been disappointing, with reported recurrence rates of 30 to 76% [10]. The effectiveness of prophylactic synthetic mesh at the time of stoma formation for the prevention of PSH also remains uncertain [10]. Hence, other types of intervention are needed to improve the QoL of people with PSH. One potential intervention is physical activity.

People with a stoma have identified PSH and physical activity as top research priorities in relation to their quality of life (QoL) [11]. Studies highlight a trend toward inactivity after stoma formation surgery, with fear of PSH being a major deterrent to being physically active [12–14]. Both patient and surgical factors influence the risk of developing PSH [15]. Patient risk factors include high body mass index (BMI) [8, 16, 17], waist circumference [18] and low levels of physical activity [19, 20]. PSH is associated with poor body image (a psychological construct that captures the perceptions, emotions, and attitudes a person holds towards his/her own body [21]), and both PSH and poor body image are associated with poor general QoL [20, 22–26] and poor physical functioning [24, 25].

### Physical activity and parastomal hernia

**Fig. 1:**
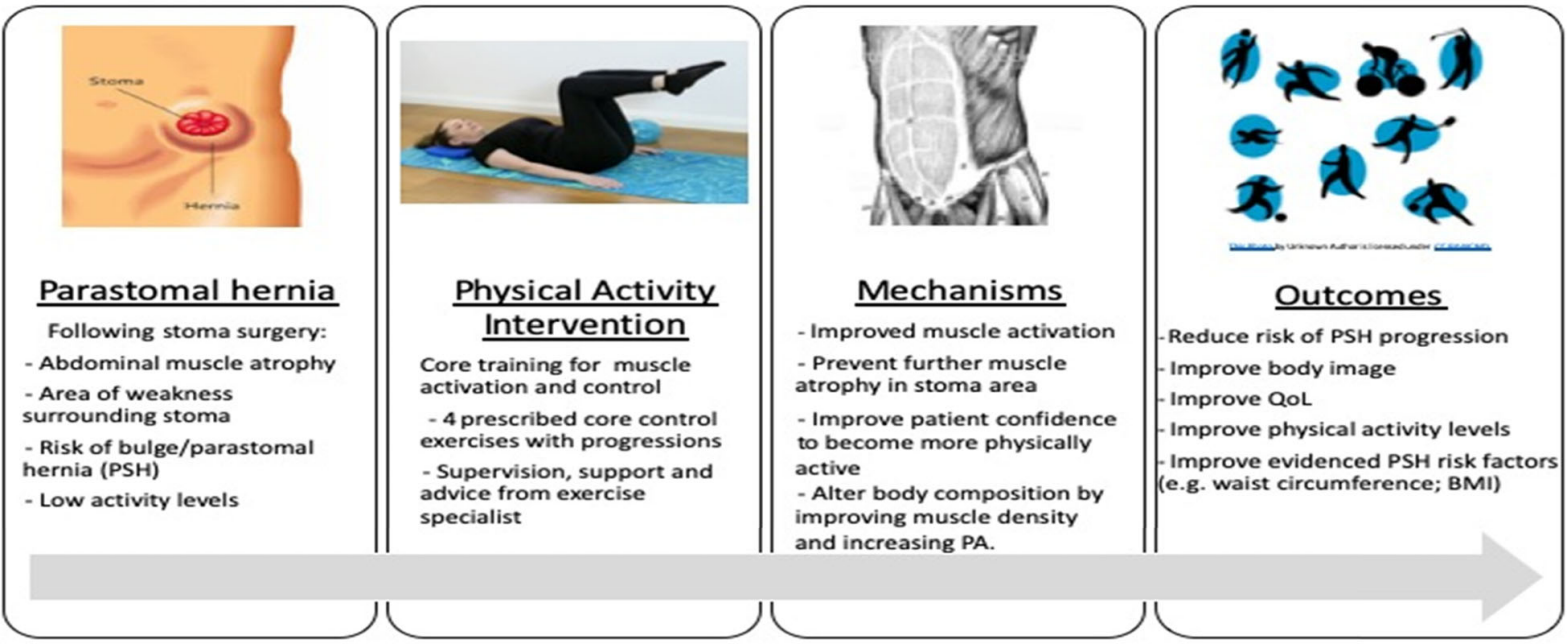
Logic model

To our knowledge, no physical activity intervention studies have been conducted that specifically target people with PSH. We hypothesise (see Fig. 1; logic model) that a physical activity intervention, that includes deep core muscle training, will elicit an adaptation response to re-engage muscle activation and control to provide an improved deep ‘corset’ support of the abdominal wall, thereby reducing the risk of PSH progression. Evidence shows that core activation leads to thickening on the abdominal muscles and a decrease in the cross-sectional area of the trunk (*p* < 0.001) [5] and that core training can lead to muscle thickening [27]. We also believe that a physical activity intervention will reduce the risk of PSH progression by effecting known risk factors for PSH such as BMI and low levels of physical activity.

There is no published research about the effect of physical activity in people with a stoma; hence, this hypothesis is based on evidence cited above as well as from systematic reviews and meta-analysis of the benefits of physical activity in the general population [28, 29]. For example, a systematic review of 10 studies (6 randomised controlled trials and 4 uncontrolled trials), involving people aged 60 to 80 years, of the effects of Pilates exercise training reported a large effect size (ES) to improve muscle strength (ES = 1.23), walking and gait performances (ES = 1.39), activities of daily living, mood states and QoL (ES = 0.94), moderate to high effect on dynamic balance (ES = 0.77), and small effects on static balance (ES = 0.34) and flexibility (ES = 0.31) [30]. A meta-analysis of 57 exercise interventions (mean age 30.4 years; standard deviation 15.35, range 10.02 to 63.40) reported that exercise resulted in improved body image from pre- to post-intervention for the intervention group compared with a control group with the largest effects evidence for adults (mean ES = 0.44) and older adults (mean ES = 033) [31].

However, before embarking on a full trial to test the hypothesis, a feasibility study of the proposed physical activity intervention and trial procedures is needed.

### Feasibility studies

The Medical Research Council framework for the development of complex interventions highlights that a key element of the development and evaluation process is feasibility work prior to assessing effectiveness [32]. It is generally recommended that feasibility studies descriptively evaluate a trial’s feasibility, acceptability and safety rather than test the effectiveness of the hypotheses of the planned main large-scale trial [33]. Feasibility studies are therefore conducted to assess key intervention and trial parameters to improve the rigor of a future full trial. In feasibility studies for instance, intervention fidelity and intervention adherence may be assessed because they can impact on statistical power and interpretation of trial results, including underestimating any efficacy in a full trial. However, a challenge for assessing these key parameters is that there is no consensus on the acceptable minimum fidelity or adherence level in trials and no standardised approach to their definition and measurement in the field of complex interventions [34, 35]. A recent review of how to define and measure adherence in studies examining older adults’ participation in exercise classes for instance, suggests adherence is defined and measured in a variety of ways with different cut-off points for indicating if adherence is successful [36].

### Aims

The aim of this feasibility study is to evaluate 4 intervention parameters—fidelity, adherence, acceptability and safety and 3 trial parameters, namely eligible patients’ consent rate, acceptability of study design (i.e., randomised controlled trial), and data availability rates for measuring the following endpoints (i.e., mechanisms and outcomes (see Fig. 1)) pre- and post-intervention:

I. Diagnosis and classification of PSH
II. Muscle activation
III. Body composition (BMI, waist circumference)
IV. Patient reported outcomes: QoL, body image and physical functioning
V. Physical activity
VI. Psychological determinants of physical activity

The aim of the future trial is to determine whether a structured physical activity intervention that includes core training and signposting to written guidance about physical activity in people who have bowel stoma and have PSH improves outcomes in comparison with signposting to written guidance only.

## Methods

### Project design

This is a feasibility study of a randomised controlled trial with 2 months follow-up, in 2 sites, with 20 participants, using mixed methods. Stage 1 involves intervention development and in stage 2, intervention and trial parameters will be assessed.

### Stage 1: intervention development

The research team have already developed a physical activity intervention for people with stoma [37] and produced an intervention manual for physical activity instructors supporting people with stoma to engage in physical activity [38]. In this study, the research team will develop new materials for people who have PSH, including videos to demonstrate exercises for re-engaging the body core and abdominal muscles and instructions on techniques and breathing for these exercises. A peer review group comprising exercise experts, physiotherapists and stoma nurse specialists will advise the research team during the development of these materials. The planned intervention is described below using the Template for Intervention Description and Replication (TIDieR) sub-headings and guidance, which is a generic template for behaviour change interventions [39]:

#### Why—theory and components

We hypothesise that a physical activity intervention that includes core training will elicit an adaptation response in the core muscles to re-engage muscle activation and control to provide an improved deep ‘corset’ support of the abdominal wall thereby reducing the risk of PSH progression. We also believe that a physical activity intervention will reduce the risk of PSH progression by effecting known risk factors for PSH, such as BMI and low levels of physical activity. The intervention is based on Self-Determination Theory [40] which focuses on maintaining the motivation to be physically active by making sure that participants have ‘autonomy’ (e.g., by offering a choice of different types of core body exercises), are ‘competent’ (e.g., by being shown how to lower intra-abdominal pressure during exercise) and experience ‘relatedness’ (e.g., by recognising their fears of worsening their bulge/PSH through exercise) [41].

#### What—materials

The exercise instructor will use the manual (i). Participants will be given materials that include videos demonstrating exercises for activating the body core and abdominal muscles and instructions on techniques and breathing for these exercises (ii). Participants will be given a biofeedback stabiliser to monitor and hence, regulate intra-abdominal pressure and risk of injury (iii).

#### What—procedures

The intervention will involve participants having 12 (1 per week) consultations with an exercise instructor. Professional associations for exercise specialists, such as the UK Chartered Institute for the Management of Sport and Physical Activity acknowledge that pre-exercise screening is an important part of the duties of an exercise specialist. Hence, the instructor will use the Physical Activity Readiness Questionnaire (PARQ) long version [42] so that she can prescribe an individualised exercise programme for each participant, taking account of medical history and current health status. Participants will be prescribed 4 different types of core training exercises with 4 levels of progression. These exercises are based on the Australian Physiotherapy and Pilates Institute methods programme [43]. Over the 12-week programme, there will be gradual progression in the prescribed frequency (number of times to perform the exercises that week), intensity (rate of exertion to perform the exercise that week) and duration (minutes to perform these exercises).

The exercises will conform with the new guidelines issued by the American College of Sports Medicine for people with an ostomy appliance [44]. Gradual progression also reduces the risk of participants over-exerting themselves with exercising and causing injury.

#### Who provides, how and where

Participants will receive a weekly physical activity consultation from an exercise instructor in-person, telephone and by video conferencing. The instructor will have as a minimum a Register of Exercise Professionals Level 4 recognised qualification (the instructor in this study for instance, has a cancer rehabilitation Level 4 qualification, and has a stoma and therefore has direct personal experience). All exercises are completed at home.

#### When and how much

The intervention is of 12 weeks duration. Each participant will receive a weekly consultation. The duration of each weekly consultation is likely to vary; based on our previous study, the average range is likely to be 15–45 min (min 5; max 120; median 35).

### Stage 2: feasibility study

#### Setting

Participants will be recruited from two hospitals in a National Health Service Trust/Board: Raigmore Hospital in NHS Highland (Scotland) and St James Hospital in Leeds Teaching Hospitals NHS Trust (England).

#### Eligibility criteria

PSH can give a person a bulge around the stoma, but some bulges do not correspond to PSH, which is an abnormal protrusion of the contents of the abdominal cavity through the abdominal wall defect created during placement of a colostomy, ileostomy or ileal conduit stoma [45]. Hence, adults 16 years+ (in the UK, for the purposes of research, people 16 years old and over are adults [46]); ≥ 3 months post stoma formation surgery for bowel disease (e.g., inflammatory bowel disease, colorectal cancer) with a colostomy or ileostomy who perceive that they have a bulge or PSH or who have a clinical diagnosis of PSH will be eligible. People who are already doing core training (e.g., Pilates, yoga) will be excluded. People with previous PSH repair will be excluded.

#### Intervention and comparator group

Participants randomly allocated to the intervention group will receive the physical activity intervention as described above and signposted to information and guidance about physical activity that are provided by UK relevant charities including Ileostomy and Internal Pouch Association and Colostomy UK. If allocated to the control group, participants will be signposted to the same guidance about physical activity.

#### Main outcome of feasibility study

The main outcome of this feasibility study is a decision by an independent Study Steering Committee to proceed to a full trial using the following traffic light system to guide decision-making (Table 1).

**Table 1:**
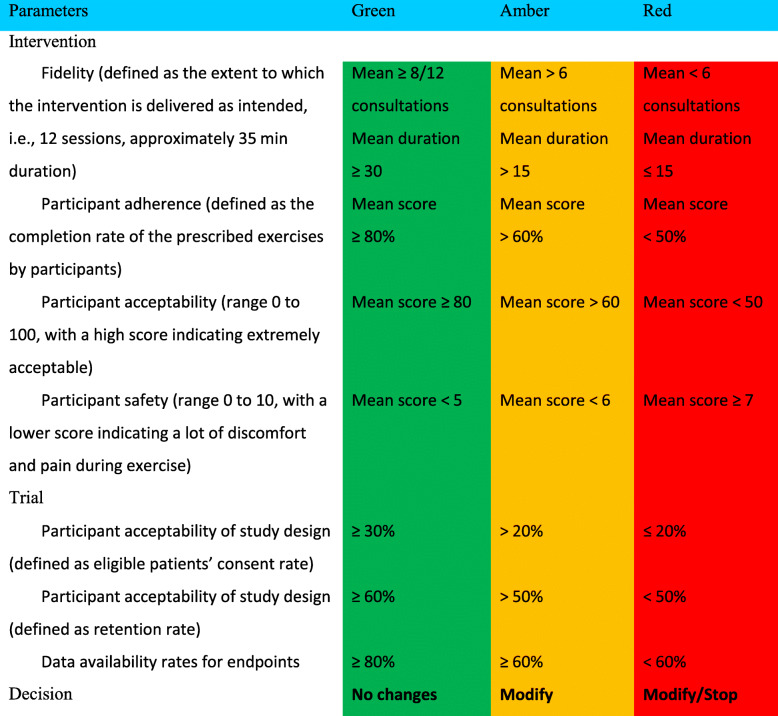
Criteria for progression from feasibility to efficacy trial

#### Intervention measures and data collection

The following measures and data collection methods will be used to assess intervention parameters:

**Intervention fidelity**

Intervention fidelity is defined as the extent to which the intervention is delivered as intended by the exercise instructor [34]. The exercise instructor will record for each participant the number of consultations, duration and medium (e.g., face-to-face; video call; telephone call). Based on our previous study of a physical activity intervention for people with stoma [47], we estimate that the mean number of consultations will be 10 and mean duration approximately 35 min (mean duration per participant over 12 weeks is 322 min). The instructor will also keep a record of the exercise prescription each week, and make summary notes throughout the 12-week programme.

**Intervention adherence**

All exercises are completed at home. Intervention adherence is defined as the completion rate of the prescribed exercises by participants [36]. Participants will write down in a physical activity diary the frequency, intensity and duration of doing the core training exercises that are prescribed by the exercise instructor in a diary that they will complete each week for the 12-week duration. They will also record other types of leisure time physical activities that they do such as running, walking and swimming. Each day of the week, participants will report each type of exercise, their rate of perceived exertion using the Borg RPE Scale [48]. If more than one exercise is done in a given day, a separate row is used to record each individual activity.

A continuous rating scale will be used to assess participants’ adherence to the prescribed exercises. At the end of each week, participants will answer the following question:

‘On a scale of 0 to 10, how successful were you at completing the prescribed exercises? When answering this question think about your success in relation to the prescribed frequency, intensity and duration. A score of 10 indicates 100% successful.’

A free text box will be included in the diary so that participants can report any challenges and barriers that they encountered while performing the prescribed exercises.

**Intervention acceptability**

At the end of the study, participants will report on a continuous rating scale of 0 to 100 if the intervention was acceptable for self-managing their bulge/PSH, with a higher score indicating that the intervention was extremely useful.

The acceptability of the intervention will also be explored at the end of the study through semi-structured face-to-face or telephone interviews (depending on participant preference) with participants. A semi-structured interview is chosen as it allows flexibility with sequencing of questions and for following up on any topics that arise naturally through discussion [49]. These interviews will last approximately 30 min and cover participants’ opinions on perceived challenges and barriers to being physically active, content of the intervention, and its perceived relevance and usefulness in self-managing bulge/PSH. These interviews will also be used to gather participants’ opinions of trial procedures.

**Intervention safety**

There is no validated patient-reported or clinically relevant measure of discomfort or pain while exercising for people with PSH. Hence, a 10-point continuous rating scale that we have developed will be used to assess participants’ level of discomfort and pain while performing the prescribed exercises. Participants are advised of the potential for post-exercise pain and discomfort in the trial documentation. At the end of each week of the 12-week programme, participants will answer the following question:

‘On a scale of 0 to 10, how much discomfort and pain did you experience around your stoma and bulge/PSH when doing the prescribed exercises? A score of 0 indicates no discomfort or pain.’

Mean discomfort/pain scores for exercises over the 12-week period will be calculated. Adverse events (AEs) will be reported as part of the ethical conduct of the study as described below.

The topic of potential discomfort and pain while exercising will be discussed with participants at in-depth interviews. In addition, as a standard part of most consultations, participants will discuss how they have found the exercises in the previous week.

#### Trial measures and data collection

The following measures and data collection methods will be used to assess trial feasibility and acceptability:

**Eligible patients’ consent rate**

We will assess eligible patients’ consent rate at each hospital site. A researcher will record the number of eligible patients who consent to the study. Based on our previous study of a physical activity intervention for people with stoma [47] and people with bowel cancer [50], we estimate a 30% consent rate.

**Acceptability of randomised controlled design**

Eligible patients’ consent rate and the retention rate for the study will be used as proxies for acceptability of a randomised controlled trial design. The retention rate is defined as the number of consenting participants who complete baseline and follow-up measures. Based on our previous study of a physical activity intervention for people with stoma [47], we estimate a 60% retention rate.

**Acceptability and data availability of outcome measures**

The acceptability of measures of outcomes will be explored in the semi-structured interviews conducted with participants at the end of the study (see above). Data availability refers to the amount of data available for analyses. In a future fully powered trial, only complete data (i.e., individually paired baseline and follow-up data for the primary outcomes) will be included in the analyses. In this feasibility study, we will therefore assess the amount of complete data for endpoints and other outcomes (Table 2).

**Table 2:** Endpoints and measures

Outcomes	Measures
Diagnosis and classification of PSH	• Clinical examination
Muscle activation	• Electromyography (EMG)
Body composition	
Waist circumference BMI	• Measuring tape • TANITA scales (weight) and stadiometer (height)
Patient reported outcomes	
QoL Body image Physical functioning	• Stoma-QoL and EQ-5D • Body image scale • Patient specific function scale
Physical activity	• Actigraph GT3X+ accelerometer
Psychological determinants of physical activity	• Basic psychological needs in exercise scale • Exercise self-efficacy

***Diagnosis and classification of bulge/PSH***

Documentation of the size of a bulge/PSH and changes over time will be assessed by clinical examination. There is no gold standard examination to assess, diagnose and classify PSH [3]. CT is highly accurate at identifying PSH but is difficult to justify because of cost, and there is risk of radiation exposure. A radiologist and medical physicist in one of the research sites had ethical concerns about increased radiation exposure, particularly in young adult participants with inflammatory bowel disease (IBD). They also were not confident that there was capacity to meet the study’s baseline and follow-up timepoints due to pressure on this resource for clinical purposes. Hence, it was decided to use clinical examination to diagnose and classify bulge/PSH. Clinical examination has sensitivity rates between 66 and 94%, and specificity rates are reported to be as high as 100% [3]. The grade of PSH will be recorded using a classification system recommended by the European Hernia Society [3, 45]. Each participant will have a clinical examination by a member of their clinical team (e.g., surgeon or nurse).

As illustrated in Fig. 1, we hypothesise that the PA intervention will lead to improved muscle activation, prevent muscle atrophy in the stoma area, and improve muscle density via activation and control. We also hypothesise that patient-level risk factors such as BMI and waist circumference will be reduced by the PA intervention.

***Muscle activation***

Any changes to muscle activation will be assessed using electromyography (EMG) to record the electrical activity of the abdominal muscles [51]. The EMG will record the electrical activation of the cells to demonstrate the level of contraction achieved.

***Body composition***

An abdominal CT scan would allow assessment of the physiological changes and body composition because it would enable calculation of abdominal muscle density and abdominal muscle/adipose tissue ratio by analysing the L3 lumbar muscle segments of each participant. Abdominal circumference measurements and assessment of abdominal adiposity can similarly be calculated. However, as described in the previous section, a decision to not use a CT scan in this study was made. The following non-invasive methods for measuring body composition will therefore be adopted instead:

Waist circumference will be measured by a researcher who will receive written and pictorial instructions on proper performance of the measurement [52]. All measurements will be performed using the MyoTape (AccuFitness, LLC) on bare skin or over thin garments. BMI is an easily calculated assessment of body weight adjusted for height (weight in kilograms/height in metres). Body weight (kg) will be measured by a researcher using TANITA scales. Patients will be asked to remove any outer garments, take off shoes and empty pockets. Stadiometers will be used to measure height (cm). Patients will be instructed to remove their shoes, stand with feet flat on the floor, feet together and heels against the wall, and with shoulder blades and buttocks also touching the wall, arms hanging loosely by their side, and facing straight ahead. They will be instructed to breathe in deeply and stretch to their fullest height when the measurement is taken. BMI will be calculated from these measurements using the standard formula of weight (kg)/height (m)^2^. BMI scores can then be translated into the following categories underweight, normal weight, overweight or obese.

***Patient reported outcomes: quality of life, body image and physical functioning***

In a future full-scale trial, we intend for QoL to be the primary outcome; this variable provides us with the patient perspective of the intervention’s direct clinical benefit and is an outcome that is considered important to patients [53]. To our knowledge, there are no bespoke instruments for assessing PSH-related QoL or for body image in this group. There are, however, generic QoL and body image instruments and several stoma-specific QoL tools [54] that we will use. PSH-specific questions will be incorporated. We will use the following instruments that will be completed by participants at baseline and follow-up. Each participant will meet with a researcher face to face to complete questionnaires hosted by Bristol On-line Survey, which is an online service that allows researchers to develop, deploy and analyse an online survey. Which instruments we use in the planned full trial will be decided by the independent Study Steering Committee (see Main outcome of feasibility section) who will draw on completion rates for each measure alongside qualitative data from participant interviews about the relevance and acceptability of each measure to make an informed decision.

The generic QoL instrument that we will use is the European Quality of Life-5 Dimensions (EQ-5D-5L), which is common measure of health-related quality of life [55]. It is divided into two sections: the EQ-5D index and the EQ thermometer. The EQ-5D index assesses health across five domains: mobility, self-care, usual activities, pain/discomfort and anxiety/depression. The EQ thermometer is a single 20-cm vertical visual analogue scales with a range of 0 to 100, where 0 is the worst and 100 is the best imaginable health and is completed by the user for their current health. Descriptive data from the five dimensions of the EQ-5D part 1 can be used to generate a health-related quality-of-life profile for the subject, created from the 1–5 scale for each question. This can be further divided into those reporting ‘problems’ or ‘no problems’, combining some of the subscales. Part 2 is scored from 0 (worst health state imaginable) to 100 (best health state imaginable). The score from part 2 can be used to track changes in health, on an individual or group level, over time. Simulation-based estimates (mean score) of the minimal important difference (MID) of the EQ-5D-5L index score in 6 countries (including England) were generally between 0.037 and 0.069, which are similar to the MID estimates of other preference-based QoL measures [56]. The MID (mean and standard deviation) for England was 0.037 ± 0.008).

Stoma-related QoL will be measured using the Stoma-QoL [57], which was deemed acceptable for use by participants in our previous study [37]. It is a 21-item questionnaire; 19 items covering the 5 domains of work/social functioning, sexual/body image, stoma function, financial concerns, and skin irritation are scored using a 5-point Likert-type frequency scale, and 2 items measure overall life satisfaction and are scored from 0 to 100, with 0 being the worst possible score and 100 being the best score. To our knowledge, no recommended MID estimates have been published for this instrument.

The body image scale [58] will be used for assessing body image. It was chosen because it has been validated in ostomy patients [59]. It is a 10-item questionnaire with items scored using a 4-point rating scale that was developed to assess the affective (e.g., feeling self-conscious), behavioural (e.g., difficulty in looking at the naked body) and cognitive (e.g., satisfaction with appearance) aspects of body image in cancer patients.

The Patient-Specific Functional Scale (PSFS) focuses on the patient’s opinion of their function in order to provide clinicians with a reliable and valid self-reported outcome measure [60]. The patient lists up to five activities that are limited by their condition (in this study, bulge/PSH) for which they are seeking treatment (in this study exercise programme). For each activity, patients use a continuous rating scale (0 to 10) with a lower score indicating that they are unable to perform the activity, to indicate the extent to which they are able to carry out the activity. The total score is the sum of the activity scores divided by the number of activities listed. It takes an average of 4 min to complete. It is used in clinical practice and research to assess if there is a meaningful change in functional status that has occurred over time. The MID has been evaluated for certain conditions and is between 2 and 3 [60].

***Physical activity***

The amount of physical activity will be objectively measured using the Actigraph GT3X+ accelerometer (Actigraph LLC, Pensacola, FL, USA) [61]. Accelerometers record movement in such a way that it can be translated into a number of different outputs, for example total step count, bouts of physical activity at specified intensities or energy expenditure. This measure will be used to examine how physical activity levels change over the course of the study. It will be worn around the wrist and measures activity counts, steps, inclinometers, and light and moderate-to-very-vigorous physical activities. Participants will be given an accelerometer that will be worn during waking hours for 7 consecutive days for 1 week at baseline and follow-up. At the end of the 7-day period, participants will return the device to the research team.

Accelerometer devices will be initialised by a researcher as follows: (1) Device recording of physical activity will be set for 7 days, with the intention to gain a minimum of 4 valid days of data for each participant; (2) the date and time when the participant is scheduled to wear the device will be set. The sample rate will be set to 30 Hz; (3) the unique participant ID will be added to the specific device.

Once the device is returned by a participant, the Actigraph software will be used to download data, as follows: (1) The unit of measurement will be set at 10-s epochs; (2) the ‘# of axis’ setting will be set to 3, and ‘steps’, ‘lux’, ‘inclinometer’ and ‘low frequency extension’ will all be selected. The Actigraph software wear-time validation will be set to meet the following criteria: (1) minimum number of valid days required = 4; (2) non-wear time will be set at > 60 min of consecutive zeros; (3) minimum number of wear hours per day required will be set at > 10 h (600 min). Commonly reported cut-points for adults will be used to differentiate physical activity intensity using Freedson et al. [61].

***Psychological determinants of physical activity***

The Basic Psychological Needs in Exercise Scale assesses [62] determinants of physical activity from the perspective of the self-determination theory, which is the theory underpinning the intervention [41]. This is an 11-item self-report questionnaire. Participants rate each item on a 5-point scale from 1 (I don’t agree at all) to 5 (I completely agree). Items assess participants’ need fulfilment for autonomy, competence and relatedness. In line with the self-determination theory, the satisfaction of these needs results in higher levels of behavioural self-determination that in turn, is reflected by higher levels of, for example, intrinsic motivation (e.g., finding exercise enjoyable) and identified regulation (e.g., considering exercise outcomes to be personally important).

#### Participant timeline

**Fig. 2:**
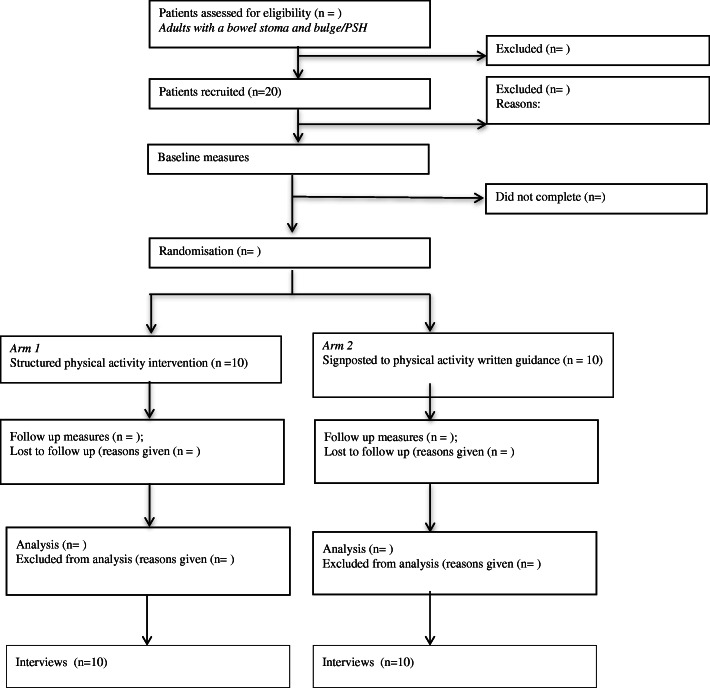
Participant flowchart

Figure 2 illustrates the process of enrolling participants in the study and timing of intervention and measurements.

#### Sample size and randomisation

The current study will confirm the feasibility of recruiting and retaining patients with stoma based on the rates obtained from our previous physical activity trial in this sample as outlined above. In a future full-scale trial, we intend for QoL to be our primary outcome; based upon the review above [30], we anticipate a large effect size. Therefore, in line with Whitehead et al., a sample size of 20 is appropriate [63].

Participants will be randomly allocated to intervention and control groups by a researcher using MinimPy (http://minimpy.sourceforge.net/introduction.html), which is a free randomisation software package to manage the process of minimizing the difference among trial groups with respect to preselected categorical factors, i.e., age and gender.

#### Recruitment and consent

Two recruitment methods that we have tested in our previous study [47] have been chosen for this study because they will maximise recruitment. The number of patients recruited by each method will be compared.

**Hospital**

A list of potentially eligible patients will be put together by the hospital clinical team who will send a letter of invitation to those that are eligible. Patients who are interested in the study will get in touch with the research team by post, telephone, or email expressing an interest in the study. A researcher will contact the potential participant by email or telephone to explain the study in more detail and confirm eligibility. Written information, in the form of a participant information sheet (PIS), will be sent to potential participants. The PIS will describe the nature of the study, what it will involve, and the known risks of taking part. Written informed consent will be obtained at a face-to-face meeting that will be arranged to coincide with the administration of baseline measures.

**Social media**

Recruitment via social media will also be used to maximise recruitment numbers. An advertisement about the study will be disseminated by members of the Patient Advisory Group and by relevant stoma charities (Ileostomy and Internal Pouch Association, Colostomy UK) on both Facebook and Twitter. Contact details of the research team will be provided, along with brief eligibility criteria. This will allow anyone who is interested in taking part to see if they will be eligible and to contact the research team directly. As above, the participant will be contacted by email or telephone and sent a PIS, and written informed consent will be obtained.

#### Data analysis

Intervention parameters (fidelity, adherence, acceptability and safety) and trial parameters (eligible patients’ consent, retention and data availability rates) will be reported overall and by site. Reasons for non-fidelity, non-adherence, non-consent, non-randomisation, and non-availability of data will be summarised. Follow-up rates will be reported overall, by site and by arm to demonstrate the acceptability of the schedule for both arms. The analysis will focus on confidence interval estimation rather than formal hypothesis testing. All outcome measures will be summarised by arm, at each time point together with 95% confidence intervals. Levels of missing data for all outcomes will be summarised overall and by arm. AEs will be monitored and rates compared descriptively between study groups. Qualitative thematic analyses of audio-recorded interviews and focus groups will be conducted using the Framework approach [64]. These data will inform the decision to proceed to a Phase III trial (Table 1).

## Ethical considerations

The research study has been approved by the North of Scotland Research Ethics Committee (REC REF 20/NS/0007).

### Consent

The ethical principles of ensuring freely given fully informed consent and the right to withdraw from research participation will apply. The right to anonymity when reporting findings will be emphasised.

### Confidentiality

All participants will be informed that all of the information that they provide to the research team will remain confidential and will only be accessible to members of that team. Only personal information that is deemed vital for running this study will be obtained. Participants will be given a unique study identifier so that their name will be filtered out of any quantitative and qualitative datasets used for analysis. The clinical exercise instructor delivering the intervention will sign a confidentiality agreement with the University of the Highlands and Islands (study sponsor), and patients will provide informed consent of their basic demographic details and relevant medical history provided for the instructor.

### Participant risk

Any exercise carries a risk of injury. To minimise this risk, the clinical exercise instructor will use the Physical Activity Readiness Questionnaire (PARQ) long version [42] so that she can prescribe an individualised exercise programme for each participant, taking account of medical history and current health status. We will ensure that the following recommendations by the UK Association of Stoma Care Nurses (ASCN) and American College of Sports Medicine (ACSM) are brought to the attention of the clinical exercise instructor delivering the PA intervention in this feasibility study. ACSN recommend core muscle exercises to strengthen the abdominis in order to prevent parastomal hernia formation. However, ASCN also advises against lifting heavy objects during stoma formation recovery since this may increase the risk of a parastomal hernia during the post-operative recovery period [65]. The new cancer and exercise American College of Sports Medicine guidelines are as follows:

- Empty ostomy bag before starting exercise.
- Weight lifting/resistance exercises should start with low resistance and progress slowly under the guidance of trained exercise professionals. People with an ostomy may be at an increased risk of parastomal hernia. To regulate intra-abdominal pressure, correct lifting technique and good form is required. Avoid use of a Valsalva maneuver.
- Modify any core exercises which cause excessive intra-abdominal pressure, namely a feeling of pressure or observed bulging of the abdomen.
- Those with an ileostomy are at increased risk of dehydration. Get medical advice on ways to maintain optimum hydration prior, during and after exercise.
- Those doing contact sports or where there is a risk of a blow to the ostomy may wish to wear an ostomy protector/shield.

### Safety reporting

The AE reporting procedures will follow those of NHS guidelines for research trials [66]. All participants will be advised and encouraged to report concerns to the research team, their stoma nurse and the clinical exercise instructor. All serious adverse events (SAE) and AEs will be recorded regardless of whether they are related to participation in the physical activity intervention and sent to the Chief Investigator who will use the NHS recommended form [66] to report the SAE to the Research and Ethical Committee that approved the study.

## Data management

Data will be kept in accordance with the Data Protection Act. The study includes four paper report forms: (i) Researchers in each site will complete a ‘recruitment form’ that will include number of eligible patients, the consent rate and reasons for declining participation; (ii) the clinical exercise instructor will complete a ‘consultation form’ indicating number, duration and type of consultation; (iii) participants will complete a diary to record prescribed activities; (iv) the study also includes the results of the clinical examination PSH classification and body composition. A researcher will remove patient names from any of these paper forms and replace with a unique identifier. Data on these paper report forms will be manually entered by a researcher into customised password encrypted spreadsheets in Microsoft Excel. A researcher will export all data entered into Microsoft Excel and Bristol Online Survey to the Statistical Package for the Social Sciences v19.0 for the purposes of analysis. All electronic data will be retained on a university password-protected server, and all paper records will be retained in a secure storage facility on university premises for a minimum of 10 years.

## Governance

The study will be conducted in accordance with the current protocol. A Study Steering Committee will provide overall supervision of the study on behalf of the study sponsor and the funder to ensure the study is conducted to the standards set out in the UK Framework for Health and Social Care Research (version 3, 2017). As noted above, the committee will make the decision whether the research team should proceed to a full trial. It will comprise senior clinicians and academics with research expertise in this area. The Study Management Group will be responsible for ensuring that the protocol is adhered to and will comprise the Chief Investigator, Researcher Assistants and the other investigators.

## Patient and public involvement

A Patient Advisory Group for a previous study [37] that included 10 people with stoma has already been involved in designing this study and will continue to be involved in this new study by, for example, developing patient information sheets and using social media to assist in recruitment.

## Discussion and dissemination

We will conduct a feasibility randomised controlled trial of a physical activity intervention in people who perceive that they have a bulge/PSH to improve QoL and other patient-reported and clinically meaningful outcomes. The feasibility and acceptability of key intervention and trial parameters will be used to decide whether to proceed to a full trial of the intervention. The findings from this feasibility study will be shared with interested parties and audiences on national and international levels. The intention of this feasibility study is to inform a full randomised controlled trial, and any outcome and finding from this preliminary work will be disseminated on that basis.

Nevertheless, we acknowledge that results from feasibility or pilot studies to estimate recruitment, randomisation and attrition rates for a full trial should be used with caution. A recent review of publicly funded trials that compared the difference in the rates between pilots and their associated full trial found high variability and therefore recommended the use of internal pilot trials [67]. Hence, we will include an internal pilot in any future trial should a decision from this feasibility study be to proceed.

To the best of our knowledge, this is the first physical activity intervention study to improve QoL in people with bowel stoma who perceive that they have PSH. The intervention, if effective, could be relevant to a large number of patients; one study reported a prevalence of PSH in up to 78%, detected either clinically or by CT [68]. Further, if the intervention halts bulge/PSH progression, then this would be clinically significant and may halt the progression of symptomatic hernias causing pain, discomfort and problems with the fitting and function of the stoma appliance [4].

We are aware of very few physical activity intervention studies involving people with stoma. Previous studies aimed to prevent PSH as opposed to supporting people to self-manage a bulge/PSH. Thompson and Trainor’s landmark studies in 2005 and 2007 evaluated an intervention that used three components—awareness of PSH, abdominal exercises, use of support belts during heavy lifting—for 1 year post-operatively to prevent a PSH [69]. They recruited a cohort in year one (*n* = 87) who did not receive the intervention and a cohort in year two (*n* = 114) and three (*n* = 99) who received the intervention, and subsequently compared PSH incidence between the three cohorts. They found PSH incidence was 28%, 14% and 17% in the year one, two and three cohorts, respectively, and found a statistically significant difference between year one and two incidences but not between years one and three [69]. They concluded that the intervention reduced PSH incidence. More recently, in a prospective quasi-experimental study, 100 patients discharged into community care from hospital were given advice to wear a lightweight support garment, given advice on lifting and hernia prevention, and given an exercise programme including three types of exercises–abdominal exercises, pelvic tiling, and knee rolling—to do five times and repeat three times a day [70]. The study found that people who developed PSH reported worse QoL than those without PSH [70]. The researchers concluded that the intervention prevented the development of PSH. However, we believe that it is not possible to draw definitive conclusions about the effect of the intervention on PSH prevention because of the methodological limitations of these studies. This proposed study aims to address these limitations by firstly developing an intervention to support people to self-manage as opposed to preventing a bulge/PSH and, secondly, using a randomised controlled study design to test the effect of a physical activity intervention on important clinical endpoints including QoL and bulge/PSH progression.

### Ethical approval and consent to participate

The research study has been approved by the North of Scotland Research Ethics Committee (REC REF 20/NS/0007).

The ethical principles of ensuring freely given fully informed consent, and the right to withdraw from research participation will apply.

## Data Availability

The datasets used and/or analysed during the current study will be available from the corresponding author on reasonable request.

## Abbreviations

BMI: Body mass index
CT: Computerised tomography
ES: Effect size
EQ-5D-5L: EuroQol five dimensions, 5 levels
MID: Minimal important difference
PSH: Parastomal hernia
QoL: Quality of life
AE: Adverse event
SEA: Serious adverse event
TiDieR: Template for Intervention Description and Replication
